# Supplementary material for: PAH deficient pathology in humanized c.1066-11G>A phenylketonuria mice
Source: Hum Mol Genet. 2024 Mar 23;33(12):1074–89. doi: 10.1093/hmg/ddae051 (PMC11153335; doi:10.1093/hmg/ddae051)

SUPPLEMENTARY MATERIAL

PAH DEFICIENT PATHOLOGY IN HUMANIZED c.1066-11G>A PHENYLKETONURIA MICE

Ainhoa Martínez-Pizarro, Sara Picó, Arístides López-Márquez, Claudia Rodriguez-López, Elena Montalvo, Mar Alvarez, Margarita Castro, Santiago Ramón-Maiques, Belén Pérez, José J Lucas, Eva Richard, Lourdes R Desviat

Supplementary Table 1. Blood serum concentrations of amino acids, whose levels were found increased/decreases in *Pah* c.1066-11A mice compared to the control WT group.

Amino acid	WT (µM)	*Pah* c.1066-11A (µM)	*p-value*
**Taurine**	360.05 ± 17.35	284.12 ± 16.17	0.0040
**Asparagine**	41.37 ± 2.31	33.62 ± 2.41	0.0293
**Proline**	58.94 ± 2.80	49.66 ± 3.25	0.0413
**Glycine**	181.2 ± 10.43	143.12 ± 7.17	0.0070
**Alanine**	328.65 ± 15.59	249.93 ± 19.37	0.0044
**Cystine**	1.31 ± 0.28	10.49 ± 1.27	0.0000
**Methionine**	48.94 ± 1.53	41.52 ± 1.69	0.0036
**Tyrosine**	68.20 ± 4.41	28.85 ± 1.66	0.0000
**Phenylalanine**	59.26 ± 2.48	1885.69 ±71.22	0.0000
**Tryptophan**	55.74 ± 3.78	42.15 ± 2.01	0.0056
**Lysine**	245.58 ± 16.57	196.64 ± 12.07	0.0267
**1-methyl-histidine**	2.54 ± 0.31	1.56 ± 0.27	0.0313
**Arginine**	106.35 ± 6.26	68.99 ± 3.95	0.0001

Concentrations are expressed as mean ± SEM; n=12 WT and n=13 PKU mice.

Supplementary Table 2. Brain concentrations of amino acids, whose levels were found increased/decreased in *Pah* c.1066-11A mice compared to the control WT group.

Amino acid	WT (µM)	*Pah* c.1066-11A (µM)	*p-value*
**Serine**	145.84 ± 4.51	218.47 ± 3.43	0.0000
**Glutamine**	1108.41 ± 46.80	822.25 ±17.70	0.0001
**Glycine**	302.5 ± 12.3	605.46 ± 15.55	0.0000
**Cystathionine**	3.13 ± 0.19	4.37 ± 0.32	0.0064
**Isoleucine**	3.71 ± 0.35	2.41 ± 0.21	0.0051
**Leucine**	13.9 ± 0.81	9.4 ± 0.39	0.0001
**Tyrosine**	14.58 ± 1.19	6.37 ± 0.33	0.0000
**Phenylalanine**	13.62 ± 0.71	153.35 ±3.94	0.0000
**Lysine**	42.18 ± 1.2	47.55 ± 1.07	0.0029
**Histidine**	12.58 ± 0.51	17.31 ± 0.46	0.0000

Concentrations are expressed as mean ± SEM; n=12 WT and n=13 PKU mice.

Supplementary Table 3. Primers used for gDNA and cDNA amplification.

Name	Sequence 5’-3	Method
**PAH 10-11-12 S**	5'-GGTAACGGAGCCAACATGGTTTACTG-3'	RT-PCR human minigene
**PAH 10-11-12 AS**	5'- AGACTCGAGGGTAGTCTATTATCTGTT-3'	
**pcDNA3.1 F**	5'-ACCCACTGCTTACTGGCTTA-3'	RT-PCR mouse minigene
**pcDNA3.1 R**	5'-GCAACTAGAAGGCACAGTCG-3'	
**hPAH exon 10 F**	5'-ACTGTGGAGTTTGGGCTCTG-3'	RT-PCR HepG2 cells
**hPAH exon 12 R**	5'-ACTGAGAAGGGCCGAGGTAT-3'	
**mPAH exon 10 F**	5’-GGCTCTTGTCATCCTTTGGA-3’	RT-PCR mice liver
**mPAH exon 11 R**	5’-TACTCCTGGCAGGCTGTCTT-3’	
**hPAH intron 10 F**	5’-ACCTATGCATGCAGCCTTGT-3’	PCR HepG2 edited cells (mutation detection)
**hPAH intron 11 R**	5’- AGGGTGGAGAACATGGGAGA-3’	
**mPAH exon 10 F**	5’-GGCTCTTGTCATCCTTTGGA-3’	PCR wt allele genotyping
**mPAH WT R**	5’-GCTTCTTCCATCTCCCCATT-3’	
**mPAH exon 10 F**	5’-GGCTCTTGTCATCCTTTGGA-3’	PCR mutant allele genotyping
**-11G>A human R**	5’-CAGTATTCCCTGCTGCATCC-3’	
**hPAH ex1-2 F**	5’-GACTTTGGACAGGAAACAAGC-3’	qPCR human PAH
**hPAH ex2-3 R**	5’-CATTCTCCTCAAATAAGCGCA-3’	
**hDNAJC12 F**	5’-CAGTGAAGACGTCAATGCACT-3’	qPCR human Dnajc12
**hDNAJC12 R**	5’-GGTTGAAGCCAGCTCCTCT-3’	
**hHSP70 F**	5’-GATCAACGACGGAGACAAGC-3’	qPCR human Hsp70
**hHSP70 R**	5’-GCTGCGAGTCGTTGAAGTAG-3’	
**mPah ex6-7 F**	5’-TTCTGCAGACTTGTACTGGT-3’	qPCR mouse PAH
**mPah ex7-8 R**	5’-ACAGATATCAGGTTCAGGTG-3’	
**mDnajc12 F**	5’-CTCTCCTCGGTTGAGCAAAT-3’	qPCR mouse Dnajc12
**mDnajc12 R**	5’-ATGCATCGAAGTTTTCACTG-3’	
**mHsp70 F**	5’-GCGCTCGAATCCTATGCCTT-3’	qPCR mouse Hsp70
**mHsp70 R**	5’-GTGCACGAACTCCTCCTTGT-3’	

**Supplementary Table 4.** DNA template, gRNA and top predicted off-targets for gene editing in HepG2 cells.					
**DNA TEMPLATE**	5′-CCTATGGGATGCAGCAGGGAATACTGATCCTGATTTAACAGTGATAATAACTTTTCACTTAGGGCCTACAGTACTGCTTATCAGAGAAGCCAAAGCTTCTCCCCCTGGAGCTGGAGAAGA-3´				
**GUIDE (gRNA)**	5′-UCUCUGAUAAGCAGUACUGU -3′				
**OFF-TARGET SEQUENCE***	**MM***	**GENOMIC LOCALIZATION**	**REGION**	**GENE**	**PRIMERS (5′-3′)**
T**G**T**AG**GA**C**AAGCAGTACTGTTGG	4	Chr8: 118873153-118873175	INTRON	SAMD12-AS1	5′-TGGTGCACTAAAGCAGCCAT-3′<br>5′-TGCCAAGGCTACGGAAAGTT-3′
TCTCT**C**A**C**A**G**GCAGTACTGTTGG	3	Chr8: 118873153-118873175	UPSTREAM	SCFD2	5′-TGGGTTCTGTTTGTTCCACC-3′<br>5′-AGTGGTTTGATAGGTGGGGG-3′
TC**AA**TGA**GG**AGCAGTACTGTGGG	4	Chr21: 39462538-39462560	EXON	GET1-SH3BGR (IKZF3)	5′-AGCAGGCACATAAGGGTGTT-3′<br>5′-GCCCACTGTCCTTAGTGGAT-3′

*mismatches (MM) shown in bold

Supplementary Table 5. DNA template, gRNA and top predicted off-targets for gene editing in mice.

**DNA TEMPLATE**	5’-AGTCCATGCTAAATGTCTCAGTTTTAGGGAATTTGCAAGAGAAGGGGCACAAATGGCCTATGGGATGCAGCAGGGAATACT GATCCTGATTTAACAGTGATAATAACTTTTCACTTGGGGCCTACAGTACTGTTTATCAGACAAGCCAAAGCTCCTGCCCC-3’				
**GUIDE (gRNA)**	5’-GUUUUAGGGAAUUUGCACUAA-3’				
**OFF-TARGET SEQUENCE***	**MM***	**GENOMIC LOCALIZATION**	**REGION**	**GENE**	**PRIMERS (5’-3’)**
**T**TT**A**TAGGGAATTTGC ACT**G**TGG	3	Chr15: 32795435-32795457	EXON	Gm32618	5’-GGGAACACCAGTTGCTACTAGC-3’<br>5’-GCACCAGAGACTCTCCAGCA-3’
**TA**T**CA**AGGGAATTTGC ACTATGG	4	Chr7: 134570396-134570418	INTRON	Dock1	5’-GGTAAAGTCCTCAGGTGGTCA-3’<br>5’-CCATGAGCCAACATTACACC-3’
GTT**A**TA**T**GGA**G**TTTGC ACTAAGG	3	Chr7: 89552521-89552543	EXON	Ccdc81	5’-CAGTGCTCTGGCTTCGTGTT-3’<br>5’-CACACACCTGCATCAAGTCA-3’
**TA**T**A**TAGGTAATTTGC ACTAAGG	4	Chr10: 120001151-120001173	INTRON	Irak3	5’-GGCAAGAGCAAGAGGAGAAGA-3’<br>5’-GAACCCAGAGCTCTCAGGTCAT-3’

*mismatches (MM) shown in bold

SUPPLEMENTARY FIGURES LEGENDS

Supplementary Figure 1. Relative mRNA expression levels of PAH and DNAJC12 and HSP70 co-chaperones. The violin plot graphs show mRNA expression analysis performed by qRT-PCR from HepG2 cells WT and -11G>A **(A)** or from liver samples of WT and PKU mice **(B)**. Statistical analysis performed by unpaired *t* test for mice and paired *t* test for cells. Solid lines represent median value and dotted lines represent quartiles. (ns, not significant).

Supplementary Figure 2. Humanized minigene and *Pah* c.1066-11A (PKU) mice. A) Schematic representation of minigene construction in the pcDNA3.1 vector. **B)** RT-PCR results after transfection in Hep3B cells of wild-type (WT) and mutant minigenes. Shown on the right is the Sanger sequencing analysis of the PCR product obtained with the mutant minigene. **C)** Gel electrophoresis of PCR genotyping in F0 mice from tail DNA biopsy. **D)** Splicing profile in liver of F5 mice by RT-PCR and Sanger sequencing from WT, Het and PKU male and female mice.

Supplementary Figure 3. BH_4 treatment. A) Administration protocol of BH_4 and **B)** Phe levels after treatment. Data are presented as mean ± SEM of n=5 BH_4 treated *Pah* c.1066-11A (PKU) mice compared to mice treated with placebo.

Supplementary Figure 4. *In silico* structural analysis of p.Q355_Y356insGLQ. A) Perpendicular views of the crystal structure of human PAH (PDB entry 6HYC) with relevant residues depicted in sticks and hydrogen bonds as dashed thin lines. To the right, modelling of the pQ355_Y356insGLQ variant, with the three inserted residues depicted in magenta. The thick dashed line indicates that the position of the residues displaced by the insertion is uncertain. **B)** Cartoon representation of the AlphaFold structural predictions for the WT protein (left) and pQ355_Y356insGLQ variant (right). Notice that the 3 amino acid insertion elongates and distorts the α-helix. In addition, the predictions in A) and B) show the steric clash between the inserted Leu residue and L333.

Supplementary Figure 1

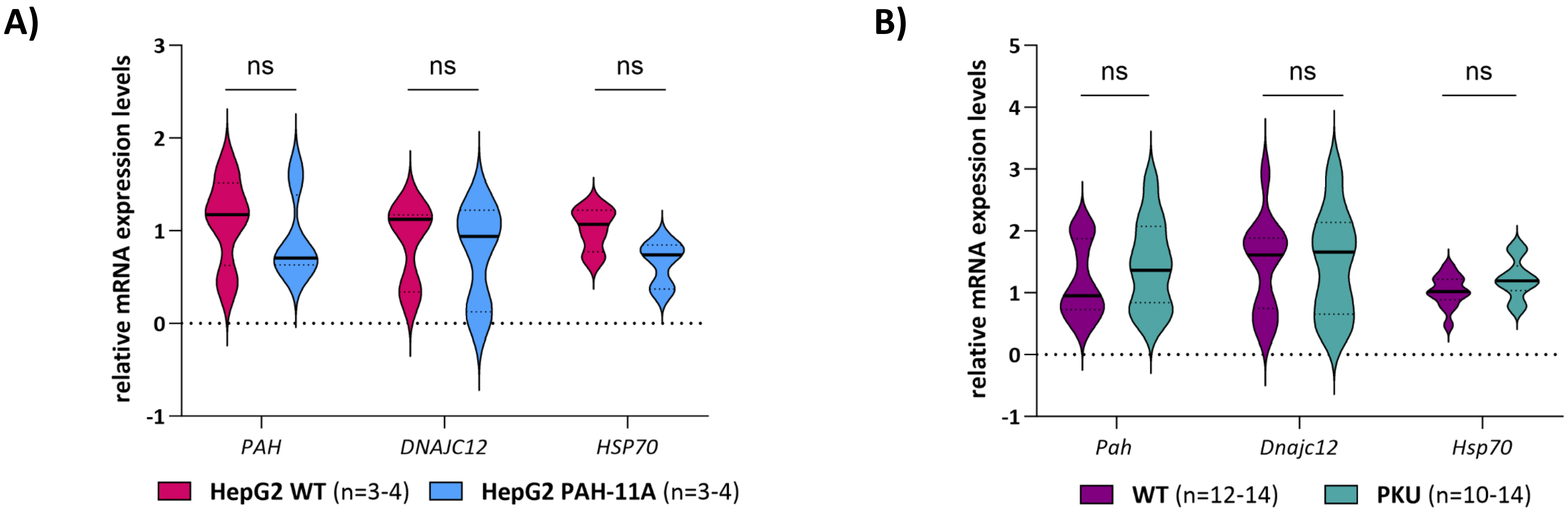

Supplementary Figure 2

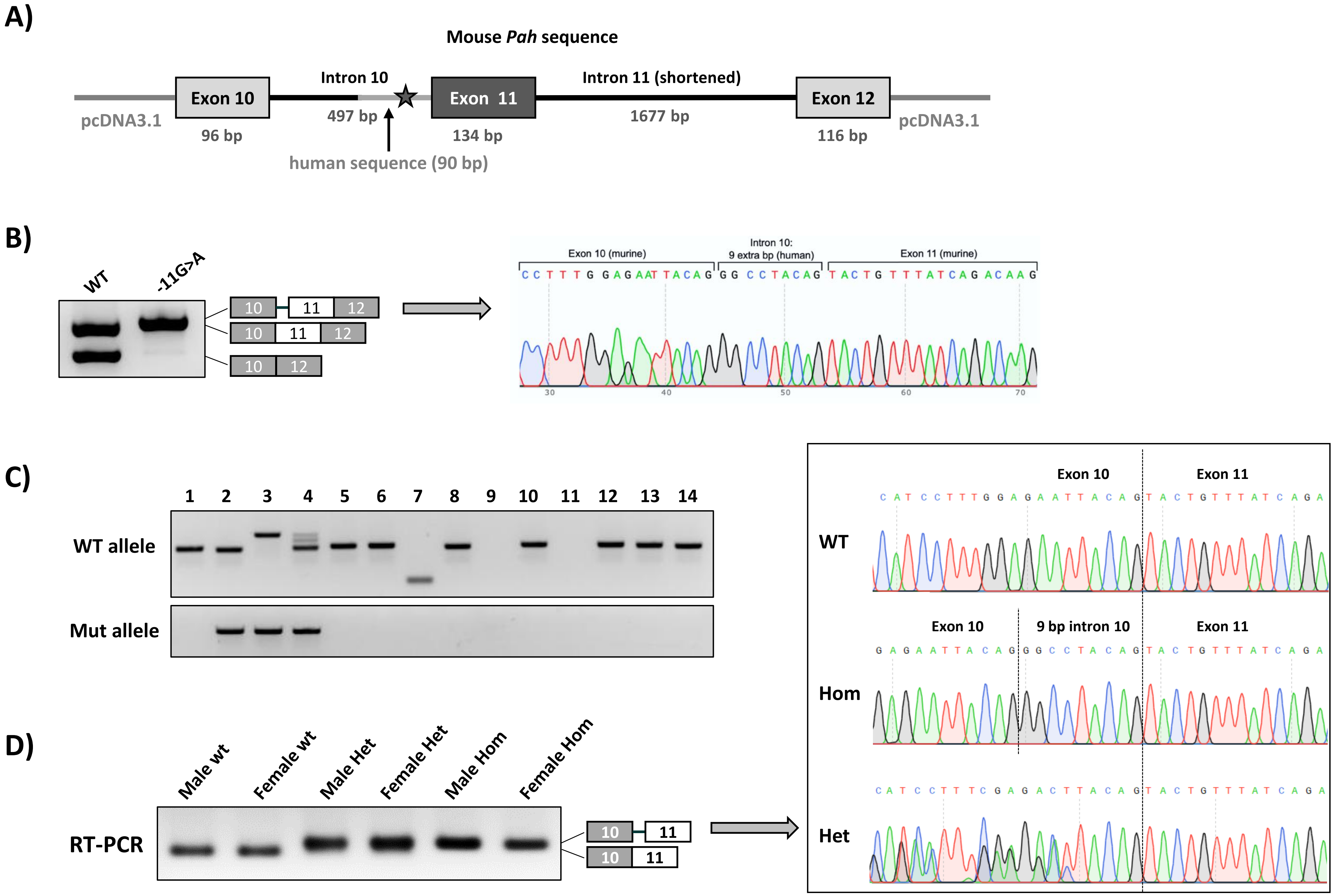

Supplementary Figure 3

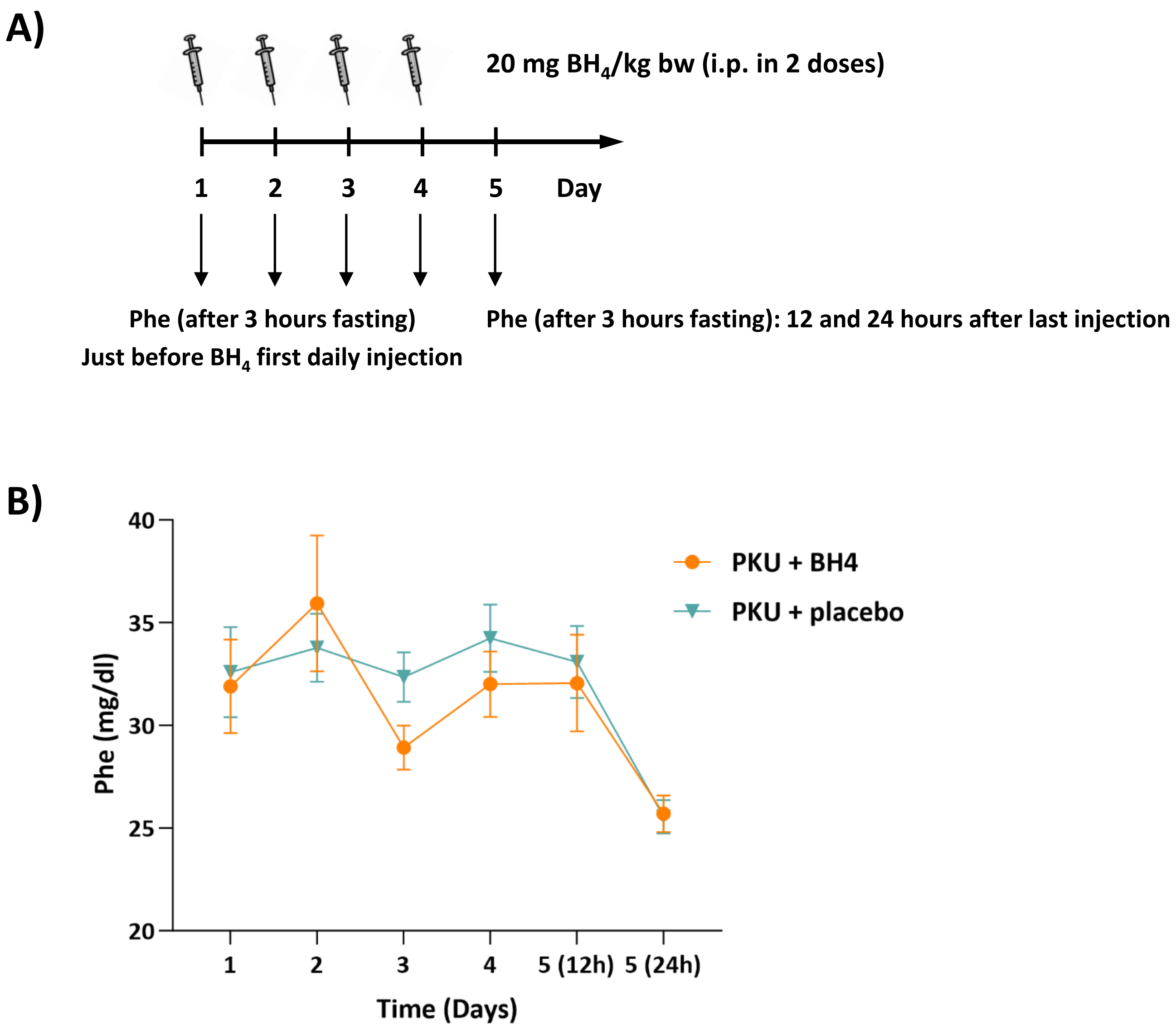

Supplementary Figure 4

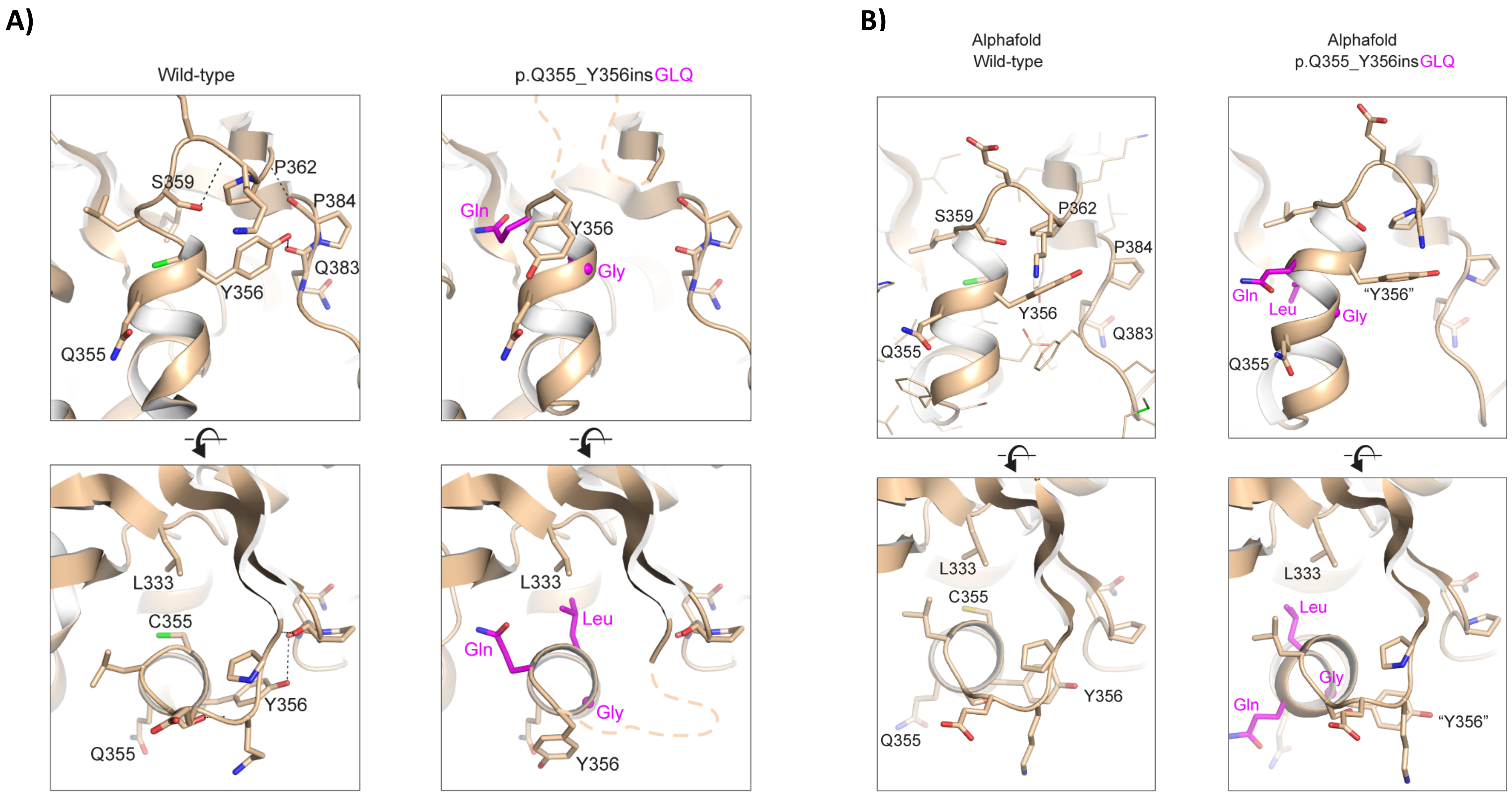

Supplement: Supplementary_material_ddae051 [file supplementary_material_ddae051.pdf]
